# An Optimized Core Distribution Adaptive Topology Reconfiguration Algorithm for NoC-Based Embedded Systems

**DOI:** 10.3390/mi16040421

**Published:** 2025-03-31

**Authors:** Bowen Hou, Dali Xu, Fangfa Fu, Bing Yang, Na Niu

**Affiliations:** 1 College of Computer and Control Engineering, Northeast Forestry University, Harbin 150040, China; 13523215706@163.com (B.H.); nefuxdl@nefu.edu.cn (D.X.); 2 Department of Microelectronics Science and Technology, Harbin Institute of Technology, Harbin 150006, China; fff1984292@hit.edu.cn; 3 Department of Software, Microelectronics School, Harbin University of Science and Technology, Harbin 150080, China; byang@hrbust.edu.cn

**Keywords:** network-on-chip, adaptive core distribution optimization, core-level redundancy, topology reconfiguration

## Abstract

In advanced multicore embedded systems, network-on-chip (NoC) is vital for core communication. With a rise in the number of cores, the incidence of core failures rises, potentially affecting system performance and stability. To address the challenges associated with core failures in network-on-chip (NoC) systems, researchers have proposed numerous topology reconfiguration algorithms. However, these algorithms fail to achieve an optimal balance between topology reconfiguration rate and recovery time. Addressing these issues, we propose an adaptive core distribution optimization topology reconfiguration algorithm, which involves the distribution of faulty cores as the main factor for the reconfiguration procedure. This algorithm is based on a 2D REmesh structure to achieve physical topology reconfiguration, optimized through a bidirectional search algorithm, and features an adaptive algorithm for optimizing core distribution. Experimental results show that a 96.70% successful reconfiguration rate with the proposed algorithm can be guaranteed when faulty cores are less than 68.75% of the max faulty cores. In particular, when the faulty cores reach 8 in the 8 × 9 REmesh, the successful reconfiguration rate is 63.60% with the proposed algorithm, which is 14.80% higher than BTTR and 9.30% higher than BSTR. Additionally, the average recovery time of our algorithm is reduced by 98.60% compared with BTTR and by 15.87% compared with BSTR, significantly improving both the performance and reliability in embedded systems.

## 1. Introduction

Recent advancements in semiconductor technology have enabled the integration of billions of transistors in very-large-scale integration (VLSI) circuits, supporting the development of sophisticated system-on-chip (SoC) architectures that unify large-scale memory arrays with programmable processing elements (PEs) [1]. However, conventional bus-based SoC designs exhibit critical limitations in scalability, latency, and power efficiency when addressing modern computational demands, ultimately hindering further performance improvements [2]. In response, the industry is transitioning towards the network-on-chip (NoC) architecture [3,4,5], a cutting-edge IC design that internalizes network communication by establishing a highly interconnected network within the chip for data exchange among processor cores, storage units, and other functional modules. NoC offers superior communication bandwidth, reduced latency, and enhanced scalability, thereby markedly improving the system’s overall performance compared to the traditional bus-sharing mechanism [6,7].

Concurrently, the rapid evolution of integrated circuit processes and modern computer technology has positioned multicore processors as a pivotal trend in computer architecture. These processors enhance computational power and parallel processing efficiency by consolidating multiple processing cores onto a single chip [8,9]. However, the increasing number of cores complicates inter-core communication and data exchange, exacerbating the challenge of core failures within the NoC—a critical component of inter-core data exchange. Core failures can severely degrade system performance and stability, making the effective management and repair of such failures a focal point of current research.

To address this challenge, researchers have introduced a range of core-level fault-tolerant techniques and algorithms, with topology reconfiguration identified as a pivotal strategy [10,11].

Topological reconfiguration refers to the dynamic modification of interconnects and computational resources within a network-on-chip (NoC) architecture to maintain functionality under faulty conditions. This process involves rerouting data paths, reallocating processing elements (PEs), or adjusting network hierarchies to circumvent both core-level failures (e.g., malfunctioning PEs or memory units) and link-level failures (e.g., broken interconnects or routing channels) [12]. Faults in NoC systems may manifest as permanent (irreversible hardware defects) or transient (temporary disturbances caused by electromagnetic interference or voltage fluctuations) [12]. Notably, the definition of “fault” remains context-dependent: while some studies refer to deadlocks and starvation as faults [13,14], some studies use stricter definitions.

Fault tolerance mechanisms extend beyond topological reconfiguration, operating concurrently at three levels:

(1) Routing Algorithms: Adaptive protocols such as self-organizing and neighbor-aware routing [12] dynamically bypass faulty nodes/links, while goal-oriented classifications optimize path selection under degraded conditions [15];
(2) Coordinate Systems: Circulant virtual coordinate frameworks decouple logical addressing from physical failures, enabling resilient rerouting independent of defective components [16];
(3) Topology Design: Architectural redundancy through spare cores or reconfigurable interconnects preemptively mitigates fault propagation [17].

A holistic fault tolerance strategy must integrate these complementary approaches to address the heterogeneous nature of NoC failures, balancing latency, energy overhead, and hardware complexity.

Core-level redundancy enhances system stability and performance by integrating spare cores into the NoC, allowing failed cores to be replaced seamlessly. Widely adopted in the industry, this approach has also become a prominent focus of research in NoC fault tolerance. Meanwhile, topology reconfiguration algorithms utilize core-level redundancy to adapt the physical topology, effectively mitigating the core failure issue in NoCs and ensuring the continued functionality of NoC systems [18].

Network-on-chip (NoC) architectures employ diverse topologies to optimize performance and scalability. Torus topologies, characterized by circular interconnects, minimize wiring complexity but exhibit constrained bisection bandwidth, limiting scalability in large-scale systems [19]. Hypercube topologies achieve logarithmic network latency through high-dimensional connectivity, yet their exponential node degree growth complicates physical implementation and power management [20]. Hierarchical topologies optimize localized communication efficiency but introduce routing overheads and resource fragmentation across abstraction layers [21]. In contrast, mesh-based topologies—notably 2D mesh and 2D REmesh topologies—have become the cornerstone of modern NoC implementations due to their structural regularity, linear scalability, and compatibility with automated electronic design automation (EDA) tools [22]. The structural homogeneity of 2D meshes facilitates seamless topology reconfiguration—a critical process for maintaining logical connectivity under core or link failures. This reconfiguration utilizes the grid-like layout of the mesh to dynamically reassign virtual-to-physical cores while maintaining communication locality, which is more difficult to achieve in irregular or high-dimensional topologies. Therefore, mesh-based topologies have obvious advantages for the realization of network-on-chip (NoC) topology reconfiguration.

In this paper, we will focus on how to find an optimized topology reconfiguration algorithm that makes the reconfigured topology arrays as similar as possible to the original arrays, while at the same time having a low time overhead and high utilization. The object of this paper is based on a 2D REmesh topology structure, and the research topic is topology reconfiguration of network-on-chip (NoC). The research objective is to improve the success rate of topology reconfiguration and enhance the performance of the algorithm by proposing an optimized topology reconfiguration algorithm, which can then be applied to a wider range of embedded systems.

This paper is structured as follows: Section 2 briefly introduces related works. Section 3 outlines the 2D mesh and 2D REmesh physical array architecture and evaluation metrics. Section 4 introduces a mathematical model to abstract the algorithm, discusses the steps in the construction of this mathematical model, describes mathematical notations, and gives mathematical proof of the optimal solution. In Section 5, we propose an optimized core distribution adaptive topology reconfiguration algorithm for NoC-based embedded systems (ACTR), describing the flow and ideas of the algorithm in detail. Experimental results and analyses are detailed in Section 6. Finally, the results of our study and the outlook of the future prospects are summarized in Section 7.

## 2. Related Works

The goal of topology reconfiguration is to determine the mapping from virtual locations to physical cores to re-establish a consistent logical connectivity for high-level applications. In recent years, researchers have proposed various optimization algorithms to address the topology reconfiguration challenge in NoCs. Zhang et al. introduced the Row-Wave Column Borrowing (RRCS) algorithm [23], which was later optimized by Yang et al. to include a bidirectional search in the local neighborhood, addressing the suboptimal solutions resulting from one-way searches [24]. Wu et al. proposed the CRS-TS algorithm, which generates an initial topology using the CRS algorithm with row double-shift and column-shift operations, followed by optimization through a forbidden search technique [25,26]. Building on these, Zheng et al. compared these algorithms and developed a two-stage topology reconfiguration algorithm that employs core-level redundancy. This approach includes a greedy strategy and an intelligent optimization method based on the memetic algorithm, both designed to refine the initial topology for improved communication performance [27].

However, although these algorithms can achieve a better reconfiguration of the logical topology, there is no guarantee that the logical topology is exactly the same as the physical topology, as the positions of the routers and cores may change throughout the topological structure. To address this issue, a 2D REmesh structure was proposed for logical topology reconfiguration. This structure guarantees consistency between the logical and physical topology, enabling complete reconfiguration of the logical topology. Furthermore, the proposed core mapping methodology, initially developed for 2D mesh architectures, demonstrates adaptability to other regular topologies through targeted structural modifications. For honeycomb configurations, axial coordinate systems and tri-directional routing protocols are essential to leverage hexagonal symmetry, though non-Manhattan interconnects introduce wiring complexity and congestion management challenges [28]. Circulant and Spidergon topologies require cyclic channel allocation and modulo-based addressing to align with circular symmetry, yet their limited path redundancy increases fault recovery latency and arbitration overhead [29,30]. While the regularity of mesh architectures inherently simplifies deterministic mappings, extending these principles to non-orthogonal topologies necessitates balancing algorithmic generality with architectural trade-offs, particularly in optimizing communication locality and fault resilience [31].

Building upon this foundation, an optimized topology reconfiguration bidirectional searching fault-tolerant algorithm for REmesh network-on-chip (BSTR) was proposed [32]. The algorithm categorizes routers and assigns connections to the corresponding cores. By employing a bidirectional search, it overcomes the limitation of the original TRADE algorithm which only considers unidirectional searches, enhancing the success rate of topology reconfiguration. Similarly, a novel topology reconfiguration backtracking algorithm for 2D REmesh networks-on-chip (BTTR) based on the same structure was proposed [33]. This algorithm considers both core allocation and router placement, utilizing recursive backtracking with pointers for searching, which enables dynamic reconfiguration with advanced fault tolerance capabilities.

However, these algorithms also have the following limitations:

(1) They do not adequately take into account the spatial distribution of faulty cores, potentially leading to suboptimal solutions;
(2) They exhibit higher computational complexity and cannot maintain a balance between the topology reconfiguration rate and the topology recovery time;
(3) They could achieve a higher resuccess rate of topology reconfiguration for low-scale, low-fault-rate topology, but it may not be applicable to high-scale, high-fault-rate topology in embedded systems.

According to the statements mentioned above, we introduce an adaptive optimized core distribution adaptive optimization topology reconfiguration algorithm (ACTR), which employs a 2D REmesh structure for physical topology reconfiguration [34,35]. Distinct from previous algorithms, our algorithm emphasizes the distribution of faulty cores and introduces an adaptive core distribution optimization algorithm. By calculating the Euclidean distance between faulty cores, we derive a core distribution factor within the sliding window to pinpoint the location of faulty cores. This distance serves as a quantitative measure to assess whether the distribution of faulty cores is concentrated or dispersed. Additionally, bidirectional search traversal order is determined by setting a threshold value, followed by the application of a sliding window algorithm to enhance algorithmic efficiency. The search order is expanded to include not only clockwise sliding along the *x*-axis but also counterclockwise sliding along the *y*-axis. Compared to the original algorithm, our algorithm has demonstrated improvements in performance metrics such as successful reconfiguration rate, average recovery time, and core reuse rate.

The contributions of this paper are summarized as follows:

(1) We propose an adaptive topology reconfiguration algorithm based on the faulty core distribution mechanism, which effectively improves the success rate of topology reconfiguration;
(2) We propose a search algorithm based on a sliding window after determining the distribution location of the faulty core, which effectively improves the efficiency of the search process and the recovery time of faulty systems.

## 3. Preliminaries

### 3.1. Two-Dimensional Mesh and 2D REmesh Architecture

**Figure 1:**
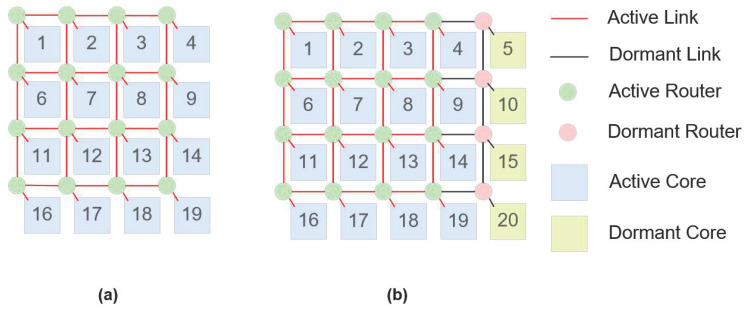
Conventional NoC architectures with and without redundancy: (**a**) 2D mesh architecture without redundancy and (**b**) 2D mesh architecture with redundancy.

Figure 1 shows a 4×4 2D mesh topology with and without one column of redundant cores. As shown in Figure 1, the 2D mesh architecture is among the simplest and most direct topologies [36]. In this configuration, each processor core is linked to the network through a router, and each router is connected to four neighboring routers. The 2D mesh topology is highly symmetrical and scalable, making it straightforward to implement in on-chip networks. However, its boundary nodes are somewhat constrained due to the limited number of connected routers, which may affect efficient network performance.

**Figure 2:**
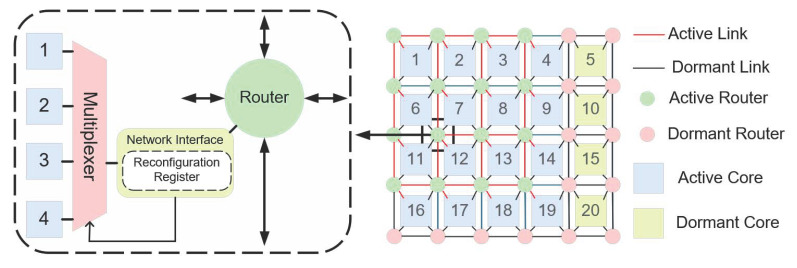
Two-Dimensional REmesh architecture with redundancy.

Figure 2 shows a 4×5 2D REmesh topology with one column of redundant cores, consisting of processor cores, on-chip routers, links, network interfaces, and multiplexers. Except for the components necessary for maintaining the normal operation of the system, all other redundant components remain in a dormant state. The 2D REmesh structure exhibits several distinctive characteristics compared to the traditional 2D mesh architecture:

(1) Constant Number of Redundant Cores: The 2D REmesh maintains the same number of redundant cores as the conventional 2D mesh, ensuring that the total area occupied by processor cores remains consistent between both structures;
(2) Addition of Routers: To enhance system scalability, the REmesh integrates an additional row and an extra column of routers atop the traditional 2D mesh. The area occupied by a single router is significantly smaller than that of a processor core, and as the network size increases, the proportion of the total area attributed to routers diminishes, rendering the area overhead associated with these additional routers acceptable;
(3) Multiplexer Connectivity: Unlike the fixed connections in a traditional 2D mesh, routers in the 2D REmesh are connected through multiplexers. This arrangement features three router types: corner routers, which connect solely to adjacent cores; edge routers, which can connect to one of two neighboring cores via multiplexers; and interior routers, which can connect to any of the four surrounding cores. By default, each router connects to the lower-right core, facilitating greater flexibility and enabling dynamic restoration of the physical topology;
(4) Enhanced Ports and Connections: The 2D REmesh structure increases overall bandwidth and mitigates data transmission bottlenecks by augmenting the number of ports and connections. This design fosters enhanced scalability, optimized load balancing, and improved flexibility, while providing robust fault tolerance and the capability for topology reconfiguration in the event of faulty cores.

Consequently, the 2D REmesh architecture can fully leverage the redundant resources of the on-chip network, thereby providing more efficient network performance.

### 3.2. REmesh Physical Array Architecture and Logical Topology Reconfiguration

**Figure 3:**
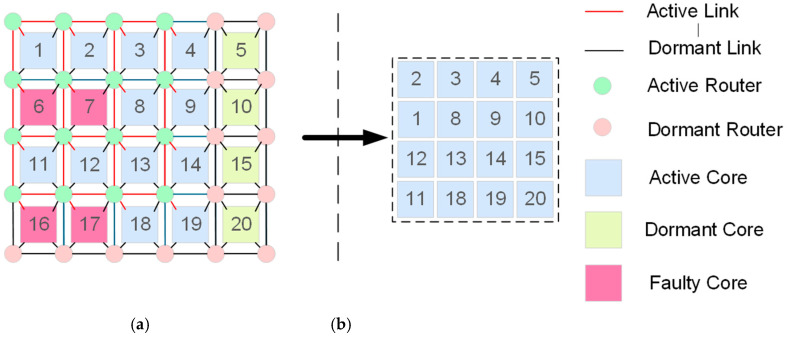
Physical array architecture and logical topology reconfiguration: (**a**) physical array architecture and (**b**) logical topology.

In an REmesh physical topology, the processor cores are located in a structure of size $m\times \left(n+k\right)$, where k denotes the number of columns of redundant cores. Figure 3 shows a typical REmesh architecture, featuring a working core size of 4×5, with an additional column of redundant cores, resulting in a network of 5×6 routers. The REmesh physical array architecture and logical topology are detailed as follows:

Figure 3a presents the REmesh physical topology, where the last column is designated for redundant cores. Within this REmesh physical architecture, each router is designed to connect with and only support one core. This design facilitates the creation of an efficient row-column formatted inter-chip interconnection network. Each box in the physical array symbolizes a core tasked with executing actual computational operations. The green circle represents the router responsible for packet forwarding and routing. The remaining rectangular boxes in different colors represent normal, faulty, and redundant cores, respectively. Figure 3b shows the REmesh logical topology of size 4 × 4 obtained by mapping Figure 3a. There are no faulty cores in this logic topology. It is obtained by replacing the faulty core in Figure 3a with a fault-free core. By using the replacement operation, the regularity and completeness of the logic topology can be guaranteed, allowing the system to work even in the presence of faulty cores.

From Figure 3, it is evident that the logical topology serves as a simplified equivalent of the physical topology. Faulty cores can be logically substituted by redundant fault-free cores located in the rightmost kth column. Since cores in the physical topology correspond to nodes within the same topology, the primary focus of this paper is on designing an efficient mapping strategy from nodes in the physical topology to those in the logical topology [35,36].

### 3.3. Evaluation Metrics

(1) Successful Reconfiguration Rate (SRR)

In the domain of REmesh topological physical architectures with dimensions $m\times \left(n+k\right)$, the objective is to accurately reconfigure the desired physical topology through logical topological manipulations. The success rate of topology reconfiguration is quantified as the ratio of successful reconfigurations to the total number of experimental tests. The mathematical formula for a successful reconfiguration rate (SRR) in this experiment is shown below:

(1) $$SRR=\frac{N_{successful}}{N_{total}}\times 100\%$$

where $N_{successful}$ represents the number of successful topology reconfigurations and $N_{total}$ represents the total number of experimental tests conducted. SRR serves as a critical benchmark for evaluating the effectiveness of topology reconfiguration algorithms. A higher SRR indicates superior algorithm performance and enhanced computational efficiency, particularly when other performance metrics remain constant.

(2) Average Recovery Time (ART)

In the domain of REmesh topological physical architectures with dimensions $m\times \left(n+k\right)$, the average recovery time is defined as the percentage of the total runtime of the topology reconfiguration algorithm relative to the total number of successful topology reconfigurations, compared to the overall rate of the experimental algorithms. The mathematical formula for average recovery time (ART) in this experiment is shown below:

(2) $$ART=\frac{T_{total}}{N_{successful}}\times 100\%$$

where $T_{total}$ represents the total runtime of the topology reconfiguration algorithm and $N_{successful}$ represents the number of successful reconfigurations. ART quantifies the time efficiency of the algorithm, with shorter ART values indicating better performance. In the actual research field of embedded systems and networks-on-chip, the time performance is often considered the key performance indicator. Therefore, the average recovery time (ART) can be used to evaluate the time performance of the topology reconfiguration algorithm.

(3) Core Reuse Rate (CRR)

In the domain of REmesh topological physical architectures with dimensions $m\times \left(n+k\right)$, the core reuse rate is defined as the percentage of original cores that have successfully undergone topology reconfiguration relative to the total number of cores in the initial array. The mathematical formula for core reuse rate (CRR) in this experiment is shown below:

(3) $$CRR=\frac{C_{reused}}{C_{total}}\times 100\%$$

where $C_{reused}$ represents the number of original cores that have successfully undergone topology reconfiguration and $C_{total}$ represents the total number of cores in the initial array. CRR highlights the effectiveness of the reconfiguration process in maintaining similarity to the original topology. A higher CRR signifies better performance and efficiency of the topology reconfiguration algorithm, particularly in system-on-chip (SoC) research.

(4) Comprehensive Evaluation Metric (CEM)

In this experiment, we innovatively propose a comprehensive evaluation metric (CEM) that considers the weighted average of the above three performance metrics and gives different metrics with different weights. The mathematical formula for the comprehensive evaluation metric (CEM) in this experiment is shown below:

(4) CEM=0.4×SRR+0.3×CRR+0.3×(1−ART)

The metrics reflect the relative importance of SRR, CRR, and ART in assessing overall algorithm performance. CEM provides a holistic view of the algorithm’s effectiveness by balancing success reconfiguration rates, core reuse rate, and time efficiency.

In this paper, we conduct experiments on different scales of network topologies based on a 2D REmesh structure, performing 1000 tests for each topology of the same scale. As the number of trials increased, the fluctuations in the successful reconfiguration rate (SRR), average recovery time (ART), core reuse rate (CRR), and comprehensive evaluation metric (CEM) decreased, leading to lower error margins and greater accuracy and reliability. Therefore, a larger experimental sample size contributes to the reduction in measurement errors and biases, thereby enhancing the reliability and accuracy of the evaluation metrics. Additionally, it strengthens the overall assessment of topology reconfiguration algorithms, which is essential for advancing research in the fields of embedded systems and network-on-chip (NoC).

## 4. Problem Formulation

### 4.1. Mathematical Model of the Proposed Algorithm

In this section, we will abstract the problem presented in the previous section and construct a mathematical model in order to describe and analyze the process of topological reconfiguration more precisely. The mathematical notations used in the mathematical models are shown in Table 1 below.

By transforming the actual problem into a mathematical expression, we are able to clearly define the scope of the problem, identify the key variables, and establish the relationship between them. This mathematical model not only helps us deeply understand the nature of the problem but also provides a solid theoretical foundation for the design and optimization of subsequent algorithms. The steps of the mathematical model are shown below:

The optimization process begins with a physical matrix $H\in R^{m\times n}$ that contains defects ***D***. A sliding window $F\in R^{p\times q}$ is used to focus on specific regions of the matrix. The process involves four main steps: Firstly, fault detection and replacement are performed to identify and mitigate defective processing elements within the matrix. Secondly, dynamic thresholds and a Quality Metric ***Q*** are used to select the appropriate optimization path. The third step involves iterative optimization, where forward and reverse searches, combined with sliding window techniques, are employed to converge towards an optimal solution. Finally, a utility function is used to compare different configurations, and a rotational search is conducted to find the most optimal target array. The output of this process is the optimized target array $T_{best}\in R^{p\times q}$.

The formulas of the mathematical model are shown as follows:

(1) The normalized Euclidean distance:
  (5) $$D_{ij}=\sqrt{{\left(\frac{x_{i}-x_{j}}{D_{max}}\right)}^{2}+{\left(\frac{y_{i}-y_{j}}{D_{max}}\right)}^{2}},\forall i,j\in F$$

In this formula, $D_{ij}$ represents the normalized Euclidean distance between points i and j, which mitigates significance loss by scaling the distance relative to the maximum distance within the array. $x_{i},x_{j},y_{i},y_{j}$ represent the coordinates of point i and point j in the physical array H. $D_{max}$ represents the maximum possible distance between any two points in the physical array, which serves as a normalization factor to ensure that all calculated distances are within a comparable range. F represents the set of pairs of faulty processing elements (PEs) within the sliding window, where i and j denote specific faulty PEs. The coordinates are typically expressed in a two-dimensional rectangular coordinate system, where $x_{i}$ and $x_{j}$ refer to the horizontal positions (or widths) of points i and j respectively. $y_{i}$ and $y_{j}$ refer to the vertical positions (or heights) of points i and j respectively.

The coordinates $x_{i}$**, $y_{i}$, $x_{j}$, $y_{j}$** are measured in nanometers (nm), reflecting the precise topological positioning of the faulty processing elements (PEs) within the integrated circuit. This unit of measurement is commonly used in modern semiconductor technologies, where the feature sizes of integrated circuits are often in the order of nanometers. Thus, using nanometers provides an accurate and relevant scale for evaluating distances and positions within the physical array H.

(2) Faulty core distribution factor within the sliding window:
  (6) $$Q=\frac{1}{t\left(t-1\right)}\sum_{i=1}^{t}\sum_{j=i+1}^{t}D_{ij}$$

In this formula, Q quantifies the spatial distribution of faulty processing elements (PEs) within the sliding window. It measures how closely or widely the faulty PEs are clustered within that given area, providing insight into density and spread. t represents the total number of faulty PEs located within the sliding window F. This variable is crucial for the normalization factor $t\left(t-1\right)$, which accounts for the combinations of point pairs.

(3) Dynamic threshold:
  (7) $$DT=\log_{10}\left(n\right)$$

In this formula, DT represents the dynamic threshold used to determine the optimization path. n represents the number of rows in a 2D REmesh structure of size $n\times \left(n+k\right)$. The base of the logarithm for the formula is **10**.

(4) Number of faulty processing elements (PEs)
  (8) $$t=\left|\left\{p_{i}\in S|p_{i}\in D\right\}\right|$$

In this formula, t represents the total number of faulty PEs, $p_{i}$ represents each processing element in the solution S. D represents the set of defective PEs. The expression inside the vertical bars $\left|\left\{\cdots \right\}\right|$ denotes the cardinality of a set, which means we are counting the number of elements in the set defined within the braces.

(5) Replacement Metric:
  (9) $$R\left(F\right)=\left|\left\{p_{i}\in F|p_{i}\in D\right\}\right|$$

In this formula, $R\left(F\right)$ represents the count of defective PEs within the sliding window F that have been replaced, $p_{i}$ represents the individual processing elements within the sliding window F.

(6) Quality Metric:
  (10) $$T\left(F\right)=\frac{1}{n_{r}}\sum_{k=1}^{n_{r}}D_{k}^{F}$$

In this formula, $T\left(F\right)$ represents the average distance to the nearest non-defective PEs within the window F. $n_{r}$ represents the number of non-defective PEs present within the sliding window F. $D_{k}^{F}$ represents the distance from each non-defective PE k located in F to the nearest defective PE.

(7) Utility Function Calculation:
  (11) $$u\left(F\right)=w_{1}\times R\left(F\right)+w_{2}\times T\left(F\right)$$

In this formula, $w_{1},w_{2}$ represents weights that denote the importance of each component in the utility calculation. The remaining mathematical symbols in this formula are shown above.

### 4.2. Mathematical Proof of the Optimal Solution

**Theorem** **1.**  The optimized algorithm achieves an optimal configuration of the target array $T_{best}$ for the given physical matrix H.

**Definition** **1.** $F_{current}$: The configuration of the sliding window before any replacements. $F_{next}$: The configuration of the sliding window after the algorithm has performed replacements on defective PEs. $T_{best}$: The optimal configuration of the target array that minimizes the utility function $u\left(F\right)$.

**Proof.**  Let $H\in R^{m\times n}$ represent the matrix containing defects in the set D. The algorithm utilizes a sliding window $F\in R^{p\times q}$ to focus on specific areas of H. In each iteration, the algorithm identifies and replaces faulty processing elements (PEs). We denote the configuration of the sliding window before replacements as $F_{current}$ and the updated configuration after replacements as $F_{next}$.

Firstly, the Replacement Metric $R\left(F\right)$ guarantees the following:

(12) $$R\left(F_{next}\right)\leq R\left(F_{current}\right)$$

Since the algorithm is designed to replace at least some defective PEs in $F_{current}$, thereby reducing the count of faulty PEs or leaving it unchanged if no further replacements can be made.

Secondly, the Quality Metric $T\left(F\right)$ guarantees the following:

(13) $$T\left(F_{next}\right)\leq T\left(F_{current}\right)$$

Since the algorithm seeks to minimize the clustering of defective PEs, resulting in a more favorable distribution in $F_{next}$.

Thirdly, the utility function $u\left(F\right)$ combines both the Replacement Metric and Quality Metric.

(14) $$u\left(F_{next}\right)\leq u\left(F_{current}\right)$$

The relationship follows from the previous inequalities, as both $R\left(F\right)$ and $Q\left(F\right)$ improve in $F_{next}$. Thus, the utility function reflects a non-increasing trend through iterations.

Finally, the iterative process continues until no further improvements can be achieved, leading to a configuration $T_{best}$ where

(15) $$u\left(T_{best}\right)={min}_{F}u\left(F\right)$$

This indicates that $T_{best}$ represents an optimal arrangement of PEs, as further iterations do not yield lower utility values. The minimization of $u\left(F\right)$ confirms that $T_{best}$ optimizes both the number of faulty PEs and their spatial distribution. Therefore, we conclude that $T_{best}$ minimizes the utility function $u\left(F\right)$.

In conclusion, the optimization algorithm conclusively achieves optimality in configuring the matrix H, supported by rigorous evaluation of relevant metrics. □

## 5. Adaptive Core Distribution Optimization Algorithm

### 5.1. ACTR Algorithm

In this section, we introduce a novel topology reconfiguration algorithm: the adaptive core distribution optimization algorithm (ACTR), which incorporates a bidirectional search for topology reconfiguration and enhances it with the addition of a faulty core distribution test and a dynamic search order adjustment to improve the performance and efficiency of the algorithm. The principal innovations of this algorithm are outlined as follows:

(1) Faulty core distribution recognition mechanism:

We propose an adaptive topology reconfiguration algorithm based on the location of the core distribution. The core of this algorithm is the examination and evaluation of the distribution of faulty cores. In the process of testing the distribution of faulty cores, we initially traverse the entire topological architecture to determine the position coordinates of each faulty core and their quantities. Subsequently, we calculate the core distribution factor by determining the normalized Euclidean distance between pairs of faulty cores. Finally, by establishing an appropriate threshold, we classify the distribution of faulty cores as either concentrated or dispersed, which then informs the final search order. In this paper, we consider three scenarios of faulty core distribution:

(a) Faulty cores that are centrally distributed in the **upper right corner** of the network, referred to as **URC** cores.
(b) Faulty cores that are centrally distributed in the **down left corner** of the network, referred to as **DLC** cores.
(c) Faulty cores that are **scattered down in the corner** of the network, referred to as **SDC** cores.

Therefore, by analyzing the distribution of faulty cores, we can approximate the optimal interval for successful topology reconfiguration and then determine different search orders based on the three cases (a), (b), and (c) mentioned above.

(2) Sliding window search mechanism:

The sliding window algorithm is an efficient algorithm for performing operations on arrays or sequences and is particularly adept at addressing problems that involve continuous subarrays or subsequences, such as identifying maximum/minimum values, sums, averages, and more. The core concept involves employing a fixed-size window that traverses the array, performing calculations within the window to avoid redundant computations, thereby enhancing algorithmic efficiency. In this paper, the sliding window is defined as a rectangular subarray containing a fixed number of cores, with the window boundaries comprising all cores that are horizontally and vertically adjacent to the window [37]. Regarding the search order, we propose a search algorithm based on a sliding window after determining the distribution location of the core. Traditional topology reconfiguration algorithms typically slide along the *x*-axis in a single direction. Specifically, the forward direction slides clockwise along the *x*-axis, while the reverse direction does the same. We introduce an additional dimension to this sliding order, namely, forward and reverse sliding in both clockwise and counterclockwise directions along the *y*-axis. Subsequently, based on the calculation results of the faulty core distribution algorithm, the following three scenarios can be obtained:

(a) If the faulty cores are centrally distributed in the upper right corner, the search order should be forward sliding clockwise along the *y*-axis and reverse sliding counterclockwise along the *x*-axis;
(b) If the faulty cores are concentrated in the lower left corner, the search sequence should be forward sliding clockwise along the *x*-axis, reverse sliding counterclockwise along the *y*-axis;
(c) If the faulty cores are scattered, the search order should be forward sliding clockwise along the *x*-axis and reverse sliding clockwise along the *x*-axis.

Therefore, the sliding window mechanism not only enhances the search efficiency of the algorithm but also, as demonstrated by comparative experiments, generates a set of optimal solutions. Experimental results demonstrate that the algorithm has good robustness and reliability.

Based on the mechanisms mentioned above for NoC-based embedded multiprocessor systems, we propose the optimized core distribution adaptive topology reconfiguration algorithm (ACTR).

The general ideas of ACTR are listed as follows:

**Step 1**: Initialize the physical array H, the router frame generated for the solution S.

**Step 2**: Locate all faulty cores in S and count the number of faulty core PEs.

**Step 3**: Calculate the normalized Euclidean distance between the faulty core PEs, generate the core distribution factor Q, and set a dynamic threshold to determine whether special treatment is needed.

**Step 4**: Based on the distribution of faulty core PEs, decide the final traversal order, which may involve comparing the number of faulty core PEs in different regions.

**Step 5**: Output the processed solution S as the final target array T.

The pseudo-code of this core algorithm is shown below (Algorithm 1).
**Algorithm 1.** ACTR Algorithm.**Input:** An $n\times \left(n+k\right)$ physical array H**Output:** An n×n target array T1: *S := H*;2: Initialize *t* := 0;3: **for** *i* := 0 to *n − 1* **do**4:  **for** *j* := 0 to *n − 1* **do**5:   Pan the routers to generate a new n×n router framework for solution S;6:  **end for**7: **end for**8: **for** *i* := 0 to *n − 1* **do**9:  **for** *j* := 0 to *n + k − 1* **do**10:    Find all faulty cores depending on the coordinates in $S_{ij}$11:    Record the number of faulty PEs;12:    **if** $S_{ij}$ is faulty then13:     *t := t + 1*;14:    **end if**15:  **end for**16: **end for**17: **fo**r *i* := 0 to *t − 1* **do**18:  **for** *j* := *i + 1* to *t − 1* **do** /*t representing the number of faulty PEs;*/19:   Calculate the normalized Euclidean distance of faulty PEs;20:  **end for**21: **end for**22: Generate the core distribution factor for Q;23: Dynamic_threshold := $\log_{10}n$;24: **if** Dynamic_threshold >= Q **then**25:  T **:= ACTR-SDC(**S**);**26:  **return** T;27: **end if**28: **for** *i* := 0 to *t − 1* **do**29:  Calculate the upper right numbers of faulty PEs in *count1*;30:  Calculate the lower left numbers of faulty PEs in *count2*;31: **end for**32: **if** *count1* ≥ *count2* **then**33:  T **:= ACTR-URC(**S**);**34: **else**35:  T **:= ACTR-DLC(**S**);**36: **end if**37: **return** T;

In order to analyze the time complexity of the ACTR algorithm, we have taken an example of the reconfiguration of a target topology of size $n\times \left(n+k\right)$. In this algorithm, the generation of the router framework involves traversing an n×n grid, resulting in a complexity of $O\left(n^{2}\right)$. Identifying faulty cores requires iterating through the physical array S of size $n\times \left(n+k\right)$, leading to a complexity of $O\left(n\left(n+k\right)\right)=O\left(n^{2}+nk\right)$. The calculation of normalized Euclidean distances among faulty processing elements (PEs) involves nested loops, yielding a worst-case complexity of $O\left(t^{2}\right)$, which can also be approximated as $O\left(n^{2}\right)$ under maximum fault conditions. Additionally, generating the core distribution factor and calculating the dynamic threshold are $O\left(1\right)$ operations. The final counting of faulty PEs contributes $O\left(n\left(n+k\right)\right)$. In summary, the total time complexity of the entire ACTR algorithm is approximated as $O\left(n^{2}+nk\right)$, emphasizing the significant impact of both n and k on performance.

ACTR involves the ACTR-URC procedure, ACTR-DLC procedure, and ACTR-SDC procedure to determine and locate the distribution of faulty cores. The general framework of these three procedures is basically the same, but the order of their search is different. The SDW procedure is invoked to perform the search. It is designed to receive an initial solution from the ACTR algorithm and eventually generate the optimal solution for the search.

The pseudo-code for one of the derived algorithms will be given below (Algorithm 2).
**Algorithm 2.** ACTR-URC Procedure.**Input:** An initial solution $S_{init}$ generated by **ACTR**;**Output:** Optimized search solution $P_{ubest}$1: $P_{init}$ := $S_{init}$2: max_iterations :=k3: iteration_count := 04: $P_{ubest}$ := $P_{init}$;5: **while** iteration_count < max_iterations **do**6:   $P_{temp}$ := **SDW(**$P_{init}$**)**;      //Update using SDW based on current solution7:   $P_{init}$ := $P_{temp}$;8:   Perform a forward search with searching order sliding clockwise along the *y*-axis;9:   Perform a reverse search with searching order sliding anticlockwise along the *x*-axis;10:    **if** Utility($P_{init}$) < Utility($P_{ubest}$) **then**      //Update only if the new configuration is better11:      $P_{ubest}$ := $P_{init}$;       //Update the best solution12:    **end if**13:    iteration_count := iteration_count + 1;14: **end while**15: **return** $P_{ubest}$;

Analyzing the time complexity of the above procedures, it can be concluded that the time complexity of the derived algorithm mainly depends on the condition of loop termination and the time complexity of the SDW procedure. Given that the SDW procedure operates with a time complexity of $O\left(m\times n\right)$, where m and n are the dimensions of the physical array H, and considering that the main loop executes for a maximum of k iterations, the overall time complexity of the ACTR-URC procedure can be expressed as $O\left(k\times m\times n\right)$. This reflects the cumulative cost of performing the SDW procedure and the subsequent forward and reverse searches within each iteration. Therefore, if k is kept within reasonable limits, the algorithm efficiently balances exploration and optimization, making it suitable for practical applications in managing configurations of faulty processing elements. In summary, the derived time complexity of the algorithm is $O\left(k\times m\times n\right)$, indicating linear scalability with respect to both the number of iterations and the size of the input array.

The SDW procedure is designed to efficiently address the issue of fault core distribution in a physical topology. The procedure aims to input a physical topology H of size m×n, including faulty cores D, and ultimately outputs a target array of size p×q after optimization. The general steps of the procedure are as follows:

**Step 1**: Initialize an empty target array $T_{best}$ to store the optimal configuration and precompute the positions of all non-defective PEs in the physical array H.

**Step 2**: Define a sliding window F of size p×q at the top-left corner of the physical array H.

**Step 3**: Traverse each possible position of the physical array H by moving the sliding window F.

**Step 4**: For each window position, identify faulty core PEs and replace them with the nearest non-defective core PEs using the precomputed positions.

**Step 5**: After replacing the defective PEs, calculate the utility value $u\left(F\right)$ based on the current configuration of the window and update $T_{best}$ if the current utility exceeds the previous best.

**Step 6**: If the target array is square, rotate the window and repeat the traversal to explore potentially better configurations.

**Step 7**: Return the optimized $T_{best}$ as the final result.

The pseudo code for this search procedure is shown below (Algorithm 3).
**Algorithm 3**. SDW Procedure.**Input:** An m×n physical array H with defect locations D**Output:** Optimized target array $T_{best}$ of size p×q1: Initialize $T_{best}$ to an empty array;2: Precompute non-defective PEs positions in H and store in a list N;3: Initialize a sliding window F of size p×q at the top-left corner of H;4: **Call** Sub-SlideWindow(H, F, $T_{best\;}$, D);5: **Procedure** Sub-SlideWindow(H, F, $T_{best}\;$, D);6: **for** each row i from 0 to m-p **do**7:   **for** each column j from 0 to n-q **do**8:     Move the sliding window F to position (i, j) in H;9:     Initialize currentUtility = 0;     //Store utility for the current window configuration10:      **for** each defect in D **do**11:       Find the nearest non-defective PEs using precomputed positions in N;12:       Replace the defective PEs with the nearest non-defective PEs;13:       Update currentUtility based on the configuration of F;14:      **end for;**15:      **if** currentUtility > $u\left(T_{best}\right)$ **then**     //Update condition corrected for maximization16:       Update $T_{best}$ to the current configuration of F;17:     **end if;**18:   **end for;**19: **end for;**20: **if** p = q **then**21:  //Optional: If the target array is square, try rotating the window;22:  **Call** Sub-SlideWindow(H, F rotated,${\;T}_{best\;}$, D);23:  //Choose the better configuration between the current $T_{best}$ and the rotated one;24:  Update $T_{best}$ to the better configuration;25: **end if;**26: **return** $T_{best}$;

In order to analyze the time complexity of the SDW procedure, we have taken a physical topology of size. In this algorithm, the sliding window traversal requires a double loop, while for each window, the algorithm needs to find the nearest non-defective PEs for each defect and replace them. It also needs to calculate the effectiveness value in order to update the value of Tbest. In summary, the total time complexity of this SDW procedure is approximated as $O\left(r\times s\times t\right)$, where r=p×q, $s=\left(m-p+1\right)$, $t=\left(n-q+1\right)$.

### 5.2. Example of Topology Reconfiguration in REmesh NoC

To delve deeper into the study of physical topology reconfiguration processes and to explore suitable algorithms for topology reconfiguration, this section provides an analysis and demonstration of a topology reconfiguration example for a 2D REmesh-based NoC network. The following figures present a feasible solution for an example of 2D REmesh topology reconfiguration.

**Figure 4:**
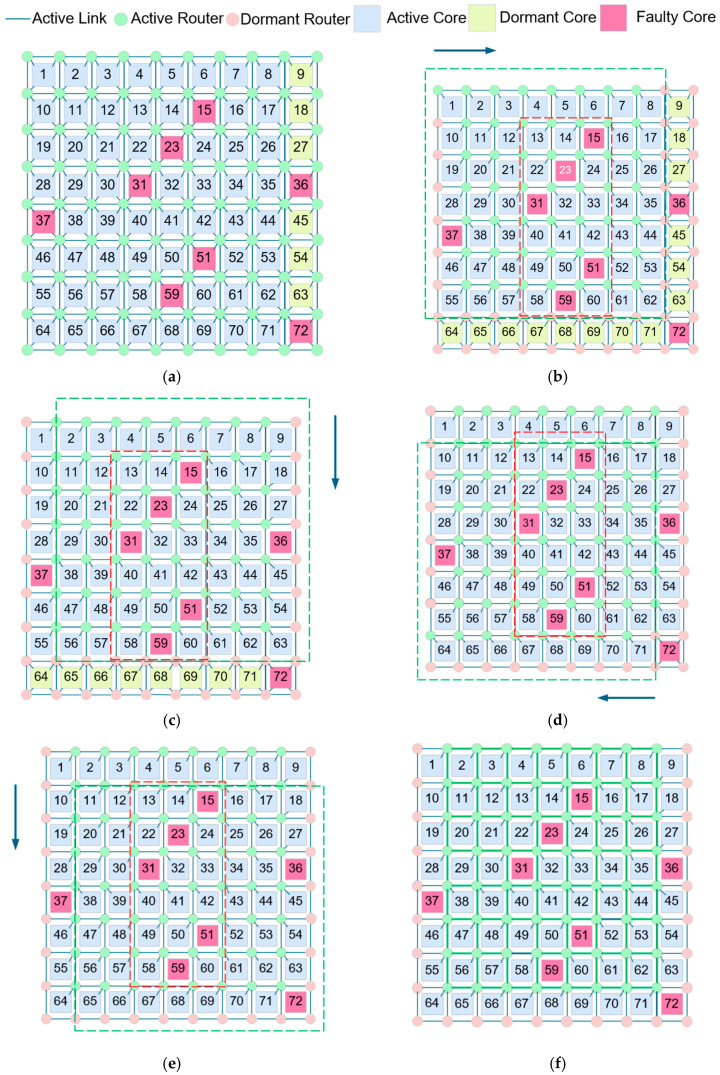
Example of topology reconfiguration in REmesh NoC: (**a**) initial topology, (**b**) upper left search for reconfigured topology, (**c**) upper right search for reconfigured topology, (**d**) lower left search for reconfigured topology, (**e**) lower right search for reconfigured topology, and (**f**) target topology.

Figure 4a presents an 8 × 8 REmesh architecture that includes one column of redundant cores. Initially, the 8 × 8 routing frame in the upper left corner serves as the default working frame, with each router individually connected to its corresponding lower right core. The cores in the rightmost column remain dormant. When a processor core failure occurs, the system identifies it as non-operational, disrupting the original regular 8 × 8 physical topology and affecting the system’s virtual topology. To reconfigure the system’s physical topology, the NoC activates redundant resources and broadcasts a topology reconfiguration message, enabling the REmesh to reestablish an 8 × 8 2D REmesh physical topology.

As shown in the figure above, cores 15, 23, 31, 36, 37, 51, 59, and 72 have experienced failures and are unable to connect to their respective routers. In this example, we use different colors to represent successfully connected routers and processor cores, as well as routers and processor cores with failed or redundant connections, and the red dashed box outlines the primary area where the failed cores are concentrated. The detailed procedure for topology reconfiguration is shown below:

**Step 1:** Initialize the sliding window size to 8×8, position it initially in the upper left corner of the entire NoC network topology. Search within this region and determine that the faulty cores are concentrated in the middle. Then, search forward and reverse directions in a clockwise direction until the entire region of the sliding window has been explored. The results are shown in Figure 4b.

**Step 2:** Move the sliding window to the right, position it in the upper right corner, and search within this region. Here, the faulty cores are concentrated in the upper half. Search the forward direction in an anticlockwise direction and the reverse direction in a clockwise direction. Update the connections between the routers and cores at this point. The results are shown in Figure 4c.

**Step 3:** Shift the sliding window downward to the lower left corner and search within this area. The faulty cores are now concentrated in the right half of the area. Search the forward direction in a clockwise direction and the reverse direction in an anticlockwise direction. Update the topology and connections accordingly. The results are shown in Figure 4d.

**Step 4:** Move the sliding window to the right, position it in the lower right corner, and repeat the previous operations. The faulty cores are concentrated in the upper half. Search the forward direction in an anticlockwise direction and the reverse direction in a clockwise direction. The results are shown in Figure 4e.

**Step 5:** Continue moving the sliding window to cover the top left, top right, bottom left, and bottom right positions until the entire NoC network has been traversed. The final updated results are shown in Figure 4f. At this point, the traversal is complete, and the area marked in green represents the correctly reconfigured 8 × 8 physical topology.

## 6. Experimental Data Results and Analysis

### 6.1. Experimental Preparation

Before introducing the experiments, it is essential to present the incentive generation module and the detection module, which are critical components of the experimental framework. These modules play a vital role in generating the necessary data for evaluating the performance of the ACTR algorithm. To assess the significance of the observed differences in performance metrics, we will employ the *T*-test for independent samples. This statistical test will provide insights into the reliability of the results, ensuring a rigorous evaluation of the algorithm’s effectiveness in reconfiguring the NoC architecture. The detailed discussion is presented as follows.

**Figure 5:**
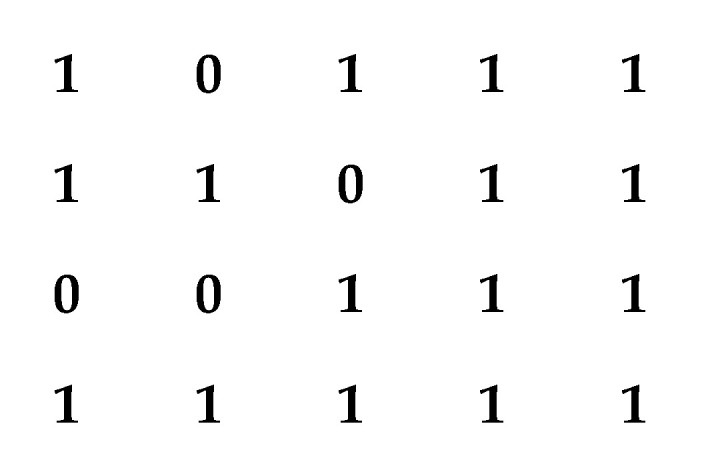
Example of the incentive generation module output.

The incentive generation module is designed to generate the states of the entire network-on-chip (NoC) architecture, simulating the operational status of each processor core—specifically, whether damage occurs during operation. This module utilizes a random number generator to randomly assign core states for various fault distributions, with the state information stored in a two-dimensional array. Each core can exist in one of two states: normal or damaged. For example, in a simulated core configuration of size 4×5 with a column of redundant cores, the module outputs a scenario where four cores are damaged. As shown in Figure 5, 1 indicates that the core is working normally, while 0 indicates that the core is damaged. The generated array provides a comprehensive view of the state of each core in the NoC, serving as the foundation for topology reconfiguration. The initial state of the randomly generated cores in this experiment is a two-dimensional matrix containing 0 s and 1 s, the same as the above example.

The detection module is used to extract non-redundant data from a large amount of randomly generated data, serving as a basis for evaluating the success rate of the algorithm with valid data. For example, in a network with a working core size of 4×5 and one column of redundant cores, there are only 20 unique scenarios when a single core is damaged. If 100,000 random injections are performed, a substantial amount of redundant data may be generated, potentially influencing the results. Consequently, our experiment incorporates a detection module to assess the redundancy among the 100,000 initial states, ensuring that only unique scenarios are considered in the evaluation of the algorithm’s performance.

The *T*-test for independent samples is employed to determine whether the means of two distinct groups are statistically different. This statistical test computes a t-statistic based on the differences between group means, the standard deviations of each group, and the respective sample sizes. A significant t-statistic indicates that the observed mean differences are unlikely to arise by chance, thus providing evidence of the algorithm’s effectiveness under various fault conditions. By assessing performance metrics such as successful reconfiguration rate (SRR) and average recovery time (ART), the *t*-test evaluates the reliability and potential advantages of the ACTR algorithm in reconfiguring the NoC topology [38,39].

The methodology for applying the *T*-test begins with data collection, wherein performance metrics are recorded across 1000 iterations for each unique initial state generated by the incentive generation module. Each iteration simulates different configurations of the NoC, ensuring a comprehensive dataset. The performance metrics are subsequently organized into groups based on predefined conditions, such as varying numbers of damaged cores. The *T*-test is then conducted to compare mean performance metrics between these groups. A significance level, typically set at α=0.05, is used to evaluate whether the observed differences are statistically significant. A *p*-value below this threshold indicates a significant difference between groups, thereby affirming the effectiveness of the ACTR algorithm in optimizing the reconfiguration of the NoC topology [40].

### 6.2. Experimental Research Plan and Methodology

In this paper, we explore REmesh network-on-chip (NoC) networks across a spectrum of sizes, with a specific emphasis on those architectures that include a column of redundant cores as the object of study. In our simulation experiments, we constructed 4×5, 8×9, 12×13, and 16×17 networks of REmeshes cores, where the redundant cores are located in the rightmost column. Our experiments systematically test the resilience to core failures by simulating the number of damaged cores ranging from 1 to the total number of redundant cores.

In our experiment, each random occurrence of faulty cores is executed 1000 times for networks of the same size, utilizing core states generated by the incentive generation module to simulate erroneous core scenarios during NoC operation. The redundancy of 100,000 initial states is examined by randomly injecting 100,000 initial states into REmesh NoCs with varying sizes and numbers of faulty cores and employing a detection module to examine the redundancy of the 100,000 initial states. The purpose of our experiment is to simulate the random distribution of faulty cores through the incentive generation module and the detection module and subsequently evaluate the topology reconfiguration of the simulated two-dimensional matrix using the ACTR algorithm, which ultimately produces a comprehensive set of performance metrics, including successful reconfiguration rate (SRR), average recovery time (ART), core reuse rate (CRR), and the comprehensive evaluation metric (CEM), which are used to evaluate the performance of the algorithm.

In this paper, our algorithm is implemented in the C programming language, and experiments are carried out on a computer equipped with a 2.5 GHz CPU and 16GB of RAM. All the algorithms in the experimental part are tested under the same fault model, that is, a unified random generator is used to generate a physical topology with randomly distributed faulty PEs. This approach ensures the robustness and credibility of the experimental results.

### 6.3. Experimental Performance Metrics Analysis

(1) Successful Reconfiguration Rate (SRR)

**Figure 6:**
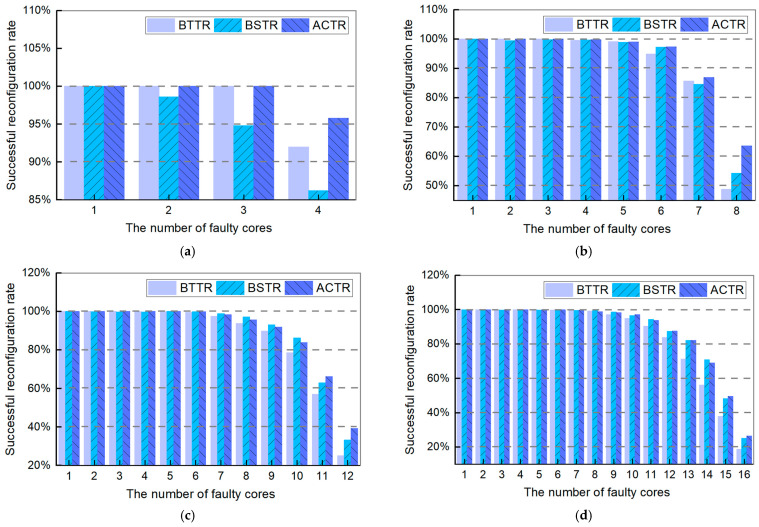
Successful reconfiguration rate of BTTR, BSTR, and ACTR in different sizes of topology networks when the number of faulty cores reaches maximum (**a**) core scale of work 4×5, (**b**) core scale of work 8×9, (**c**) core scale of work 12×13, and (**d**) core scale of work 16×17.

Figure 6 shows the comparison results of three topology reconfiguration algorithms for different network sizes with one column of redundant cores. It can be seen from the figure that as the number of faulty cores increases and the network core size increases, the overall trend of the topology reconfiguration success rate for these three algorithms is similar. Specifically, when the number of faulty cores is less than 50% of the maximum number of redundant cores, the success rate of topology reconfiguration can reach almost 100%. However, when the number of faulty cores exceeds half of the maximum number of redundant cores, the success rate of topology reconfiguration declines significantly. Nevertheless, as the number of cores and the network size increase, the topology reconfiguration success rate of our proposed adaptive topology reconfiguration algorithm is significantly better than the other two algorithms. In particular, when the number of faulty cores is less than 68.75% of the maximum number of redundant cores, the success rate of topology reconfiguration with our algorithm can reach almost 96.70%. Furthermore, our algorithm demonstrates excellent performance in terms of a successful reconfiguration rate in large-scale networks. For example, when the faulty cores reach 8 in the 8×9 REmesh, the topology reconfiguration success rate of our algorithm is 63.60%, which is 14.80% higher than BTTR and 9.30% higher than BSTR; when the faulty cores reach 12 in the 12×13 REmesh, the topology reconfiguration success rate of our algorithm is 39.30%, which is 14.30% higher than BTTR and 6.10% higher than BSTR. In particular, the successful reconfiguration rate increases with the proportion of faulty cores.

**Figure 7:**
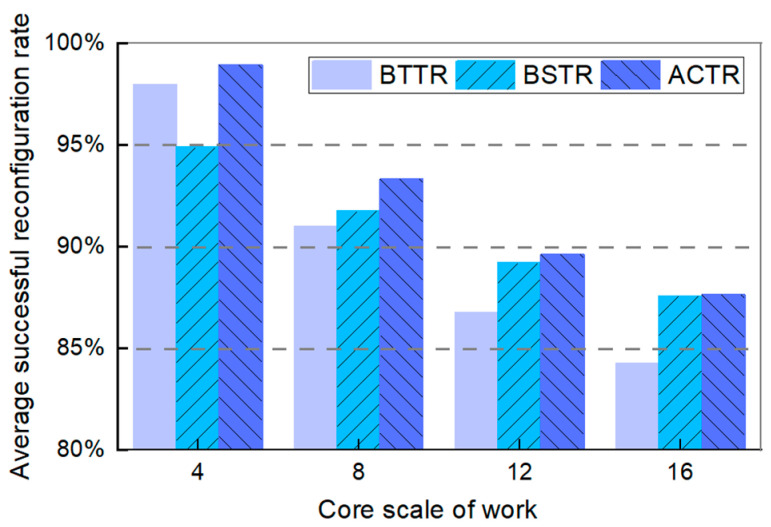
Average successful reconfiguration rate of BTTR, BSTR, and ACTR in different sizes of topology networks.

Figure 7 shows the comparison results of the average success rate of topology reconfiguration for three topology reconfiguration algorithms. As can be seen from the figure, the average success rate of topology reconfiguration of our algorithm is higher than the other two algorithms, particularly at network core sizes of 4 and 8. For example, when the working core size is 4×4, our algorithm is 4.05% higher than BSTR, and when the working core size is 8×8, our algorithm is 2.32% higher than BTTR.

Therefore, combining the graphs and data, it is concluded that the adaptive core distribution optimization algorithm can significantly improve the topology reconfiguration success rate.

(2) Core Reuse Rate (CRR)

**Figure 8:**
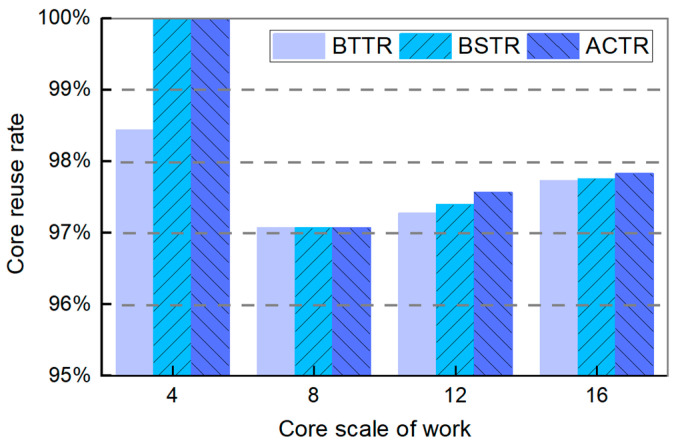
Core reuse rate of BTTR, BSTR, and ACTR in different sizes of topology networks.

Figure 8 shows the comparison results of the core reuse rate of three topology reconfiguration algorithms. As can be seen from the figure, the core reuse rate of all three algorithms exceeds 95%, indicating a high core reuse rate. Meanwhile, as the network core size increases, the core reuse rate of all three algorithms remains around 97%, with minimal differences observed. However, in a comprehensive view, the overall core reuse rate of our algorithm is still better than the other two algorithms. In particular, when the working core size is 4×4, the core reuse rate of our algorithm is 100%, which is equal to BSTR, 1.56% higher than BTTR.

(3) Average Recovery Time (ART)

**Figure 9:**
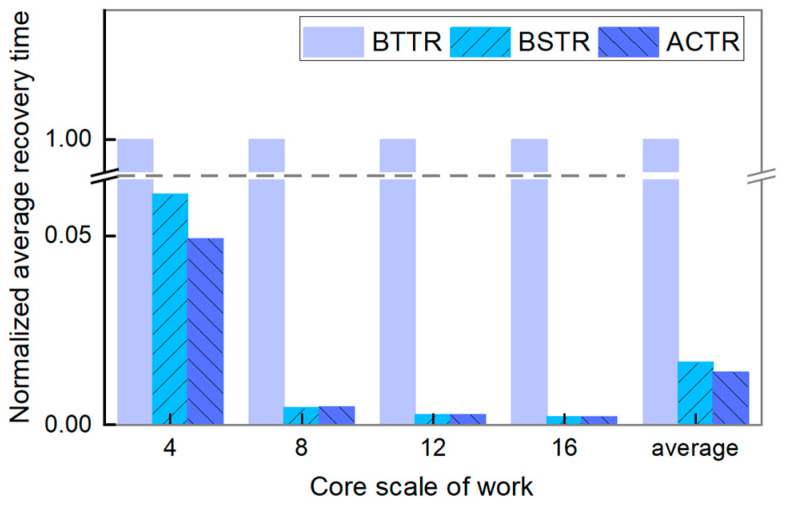
Normalized average recovery time of BTTR, BSTR, and ACTR in different sizes of topology networks.

Figure 9 shows the comparison results of the normalized average recovery time of three topology reconfiguration algorithms. As illustrated in the figure, the normalized average recovery time of BTTR is set to 1.00, since the average recovery time for BTTR is significantly longer compared to the other algorithms, resulting in a notably higher running time for the procedure. This discrepancy may be attributed to the algorithm’s extensive use of recursion and pointers, leading to a large number of recursion layers, thereby significantly increasing the running time. In contrast, the average recovery time for our algorithm is significantly better than BTTR and is comparable to BSTR, particularly at large scales. The experimental results show that the average recovery time of our algorithm is reduced by 98.60% compared with BTTR and by 15.87% compared with BSTR. Therefore, our algorithm demonstrates a superior time efficiency advantage.

(4) Comprehensive Evaluation Metric (CEM)

**Figure 10:**
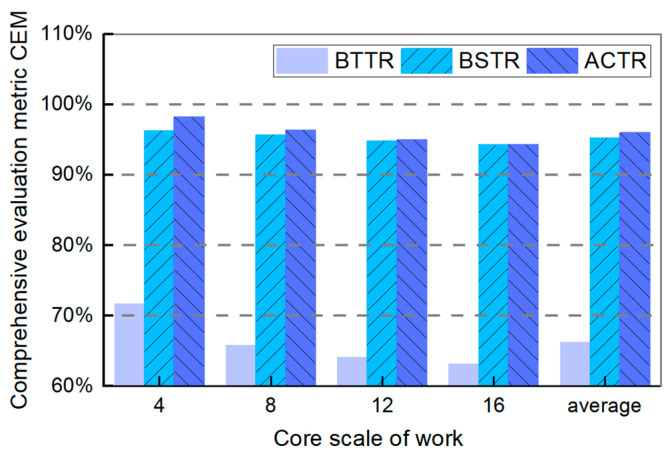
Comprehensive evaluation metric CEM of BTTR, BSTR, and ACTR in different sizes of topology networks.

In this paper, we propose a comprehensive evaluation metric CEM in order to assess the overall performance of the algorithms. The comparative results of CEM are shown below:

Figure 10 shows the comparison results of the CEM of three topology reconfiguration algorithms. As shown in the figure, the CEM of BTTR is significantly lower than the other two algorithms. In contrast, both our algorithm and BSTR achieve CEM values exceeding 90%, with our algorithm demonstrating a higher CEM. Additionally, the CEM of all three algorithms decreases gradually as the working size increases. In particular, when the working size is 4, the CEM of our algorithm is 1.94% higher than BSTR, and when the working size is 16, the CEM of our algorithm is 31.18% higher than BTTR. Furthermore, the average CEM of our algorithm is 29.80% higher than BTTR and 0.70% higher than BSTR.

In summary, the combination of the above graphs and data demonstrates that the overall CEM of our algorithm is optimal and its overall performance is superior, which indicates that our adaptive core distribution optimization algorithm achieves a higher topology reconfiguration success rate, a core reuse rate, and a shorter average recovery time. Consequently, it is a robust and efficient solution for maintaining network integrity in the presence of core failures.

## 7. Conclusions

In this paper, we have innovatively proposed an adaptive core distribution optimization algorithm combined with a faulty core distribution recognition mechanism and a sliding window search mechanism. Compared to the BTTR and BSTR, our algorithm significantly improves the success rate of topology reconfiguration as well as core reuse rate and reduces average recovery time, especially in large-scale networks. The experimental results show that a 96.70% successful reconfiguration rate with the proposed algorithm can be guaranteed when faulty cores are less than 68.75% of the max faulty cores. In particular, when the faulty cores reach 8 in the 8×9 REmesh, the successful reconfiguration rate is 63.60% with the proposed algorithm, which is 14.80% higher than BTTR and 9.30% higher than BSTR. Additionally, the average recovery time of our algorithm is reduced by 98.60% compared with BTTR and by 15.87% compared with BSTR. Therefore, our algorithm is suitable for application in large-scale networks, and future research can be extended to topology reconfiguration in large-scale networks.

## Figures and Tables

**Table 1:** The mathematical notations.

Category	Notations	Description
Input Parameters	$H\in R^{m\times n}$	The original physical array
	$F\in R^{p\times q}$	The sliding window used for searching and optimizing
	D	Set of defect locations in H
	n,k,t	Dimensions and number of faulty elements
Output Parameters	$T_{best}\in R^{p\times q}$	Final target array that is optimized

## Data Availability

The article presents the original contributions of the study. Should further discussion or questions arise, the corresponding author can be contacted directly.
